# Interaction between *Pseudomonas aeruginosa* and *Aspergillus fumigatus* in cystic fibrosis

**DOI:** 10.7717/peerj.5931

**Published:** 2018-11-09

**Authors:** Jingming Zhao, Wencheng Yu

**Affiliations:** Department of Respiratory Medicine, The Affiliated Hospital of Qingdao University, Qingdao, Shandong, China

**Keywords:** *Pseudomonas aeruginosa*, Infection, *Aspergillus fumigatus*, Intermicrobial interaction, Cystic fibrosis

## Abstract

**Background:**

Cystic fibrosis (CF) is a disease characterized by chronic airway infection with a high incidence and poor prognosis. *Pseudomonas aeruginosa* and *Aspergillus fumigatus* are pathogens commonly found in CF patients. Clinically, these two microorganisms often coexist in the airway of CF patients. Combined infection with *P. aeruginosa* and *A. fumigatus* results in worsening lung function and clinical condition.

**Methods:**

In this review, we focus on the mutual inhibition and promotion mechanisms of *P. aeruginosa* and *A. fumigatus* in CF patients. We also summarized the mechanisms of the interaction between these pathogenic microorganisms.

**Results:**

*P. aeruginosa* inhibits *A. fumigatus* growth through the effects of phenazines, the quorum sensing system, iron competition, bacteriophages, and small colony variants. *P. aeruginosa* induces *A. fumigatus* growth through volatile organic compounds and subbacteriostatic concentrations of phenazines. *A. fumigatus* interferes with *P. aeruginosa*, affecting its metabolic growth via phenazine metabolic transformation, gliotoxin production, and reduced antibiotic sensitivity.

**Discussion:**

Coexistence of *P. aeruginosa* and *A. fumigatus* can lead to both mutual inhibition and promotion. In different stages of CF disease, the interaction between these two pathogenic microorganisms may shift between promotion and inhibition. A discussion of the mechanisms of *P. aeruginosa* and *A. fumigatus* interaction can be beneficial for further treatment of CF patients and for improving the prognosis of the disease.

## Introduction

Cystic fibrosis (CF) is the most common inherited lung infection disease; it is estimated that more than 70,000 people worldwide suffer from CF (Cystic Fibrosis Foundation, 2017). As CF affects multiple organs, the morbidity and mortality of CF are caused by airway infection and the associated inflammation (Zhao et al., 2012). Mutations in the cystic fibrosis transmembrane conductance regulator (CFTR) gene result in dysfunction or a lack of CFTR protein and impaired mucociliary clearance in CF patients. CF-related lung disease begins early in life, with inflammation, impaired mucociliary clearance, and consequent chronic infection of the airways (Robinson & Bye, 2002).

The pathogens *Pseudomonas aeruginosa* and *Aspergillus fumigatus* are common in lung infections. The bacterium *P. aeruginosa* infects 70–80% of adult patients with CF (Al-Momani et al., 2016; Salsgiver et al., 2016) and *A. fumigatus* is the most common fungal pathogen isolated from the airways of CF patients. The reported prevalence of *A. fumigatus* colonization in CF patients is between 16% and 58% (Amin et al., 2010; Becker et al., 1996; Skov et al., 2005; Stevens et al., 2003; Valenza et al., 2008). Moreover, many studies have shown that the sputum of CF patients contains both *P. aeruginosa* and *A. fumigatus*. Previous studies have reported the isolation of *A. fumigatus* in up to 60% of CF patients with *P. aeruginosa* infection, and *P. aeruginosa* has been isolated in up to 64.2% of CF patients with *A. fumigatus* infection (Bakare et al., 2003; Paugam et al., 2010). The results from a systematic review and meta-analysis showed that the pooled co-colonization prevalence of *P. aeruginosa* and *A. fumigatus* in patients with CF was 15.8% (95% CI [9.9–21.8]) with variation ranging between 2.3% and 44.8% (Zhao et al., 2018).

Co-colonization by *P. aeruginosa* and *A. fumigatus* in CF patients correlates with a worsened condition (Amin et al., 2010; Shoseyov et al., 2006). For example, an Irish registry analysis showed that *P. aeruginosa* and *A. fumigatus* co-colonization was associated with reduced FEV_1_, more frequent hospitalization, greater respiratory exacerbation, and increased use of anti-microbials compared with patients without the co-existence of these pathogens (Reece et al., 2017). Another study reported increased levels of toxic products in supernatants from *P. aeruginosa* and *A. fumigatus* co-culture compared with those from *P. aeruginosa* monoculture. Indeed, the production of cytotoxic elastase by *P. aeruginosa* increases in the presence of the filamentous fungus *A. fumigatus*, damaging human lung epithelial cells, decreasing lung function and facilitating disease progression (Smith et al., 2015).

*Pseudomonas aeruginosa* and *A. fumigatus* interact in a complex manner in the airways of co-infected CF patients. In this review, we summarize in detail the mechanisms underlying the interaction between *P. aeruginosa* and *A. fumigatus*. We review the principles of mutual inhibition and growth promotion of *P. aeruginosa* and *A. fumigatus* as well as interaction between the two microorganisms in CF patients at different stages of the disease, emphasizing the impact of such interactions on the conditions of CF patients. In the presence of co-infection, *P. aeruginosa* and *A. fumigatus* do not exist in isolation; instead, they affect each other and combat the immune response together to collaboratively affect the development of the disease.

### Survey methodology

The EmBase, PubMed, and Web of Science databases were searched (until January 2018) using the following free-text terms: *P. aeruginosa*, *A. fumigatus*, and CF.

#### Inhibitory effect of P. aeruginosa on A. fumigatus

*Pseudomonas aeruginosa* inhibits *A. fumigatus* growth by the effect of phenazines, the quorum sensing (QS) system, iron competition, bacteriophages, and small colony variants (SCVs).

Phenazines constitute a large proportion of the numerous molecules secreted by *P. aeruginosa* during growth and are considered important virulence factors against target organisms, including other bacteria, fungi, and mammalian cells (Gibson, Sood & Hogan, 2009; Lau et al., 2004; Price-Whelan, Dietrich & Newman, 2006; Whiteson et al., 2014). Phenazines are present in CF patient sputum at concentrations ranging from 1 to 100 μg ml^−1^ (Wilson et al., 1988), and their increasing concentrations can cause a concomitant decline in lung function (Hunter et al., 2012). In CF patients, overproduction of alginate in *P. aeruginosa* biofilms generates a hypoxic gradient and anaerobic environment that enhances phenazine toxicity (Wang, Kern & Newman, 2010). *P. aeruginosa* phenazines have an important impact on electron shuttling, redox chemistry, and biofilm development through the toxic superoxide signaling and generation (Pierson & Pierson, 2010; Price-Whelan, Dietrich & Newman, 2006). Phenazines are regarded as endogenous redox-active molecules that promote *P. aeruginosa* growth and survival under iron-limiting conditions in CF patients and include five secreted molecules: pyocyanin (5-*N*-methyl-1-hydroxyphenazine, PYO) (Blyth & Forey, 1971; Kerr et al., 1999; Mangan, 1969), 1-hydroxyphenazine (1-HP) (Kerr et al., 1999; Mangan, 1969), phenazine-1-carboxamide (PCN), phenazine-1-carboxylic acid (PCA) (Briard et al., 2015), and dirhamnolipids (diRhls) (Briard et al., 2017).

The QS system comprises a cell density-based intercellular communication system in which signals are transmitted within the same bacterial species and between different species. The QS system regulates a variety of biological characteristics, including the release of virulence factors. The QS system in *P. aeruginosa* is involved in the regulation of elastase, pyocyanin, proteolytic enzyme, and biofilm formation (Lee & Zhang, 2015). There are three known QS systems in *P. aeruginosa*, namely, las, rhl, and pqs.

Fe is a very important element for *P. aeruginosa* and *A. fumigatus* growth. In fact, the numerous iron acquisition systems underlie the ability of *P. aeruginosa* to survive in diverse environments, with a strong ability to compete with other organisms for this essential metallonutrient. There are three classes of pyoverdines, which are iron chelators, with similar iron-binding properties and levels of activity. Type II pyoverdine is the main type involved in *P. aeruginosa* strains associated with CF (De Vos et al., 2001).

Bacteriophages have an important impact on bacterial virulence and phenotypic variation. It has been shown that the formation of SCVs in biofilms can be mediated by the filamentous bacteriophage Pf4 of the *P. aeruginosa* strain PAO1 (Mooij et al., 2007), and this morphological type is related to parameters of poor lung function in CF patients.

As SCVs represent a CF *P. aeruginosa* phenotype, analysis of SCVs isolated during chronic *P. aeruginosa* colonization in CF patients is a worthy endeavor. For example, it has been reported that SCVs isolated from CF patients are resistant to antibiotics and are associated with poor lung function and a poor clinical condition (Evans, 2015; Hogardt & Heesemann, 2010; Häussler et al., 1999).

##### Inhibitory effect of phenazines on the growth of A. fumigatus

**Figure 1:**
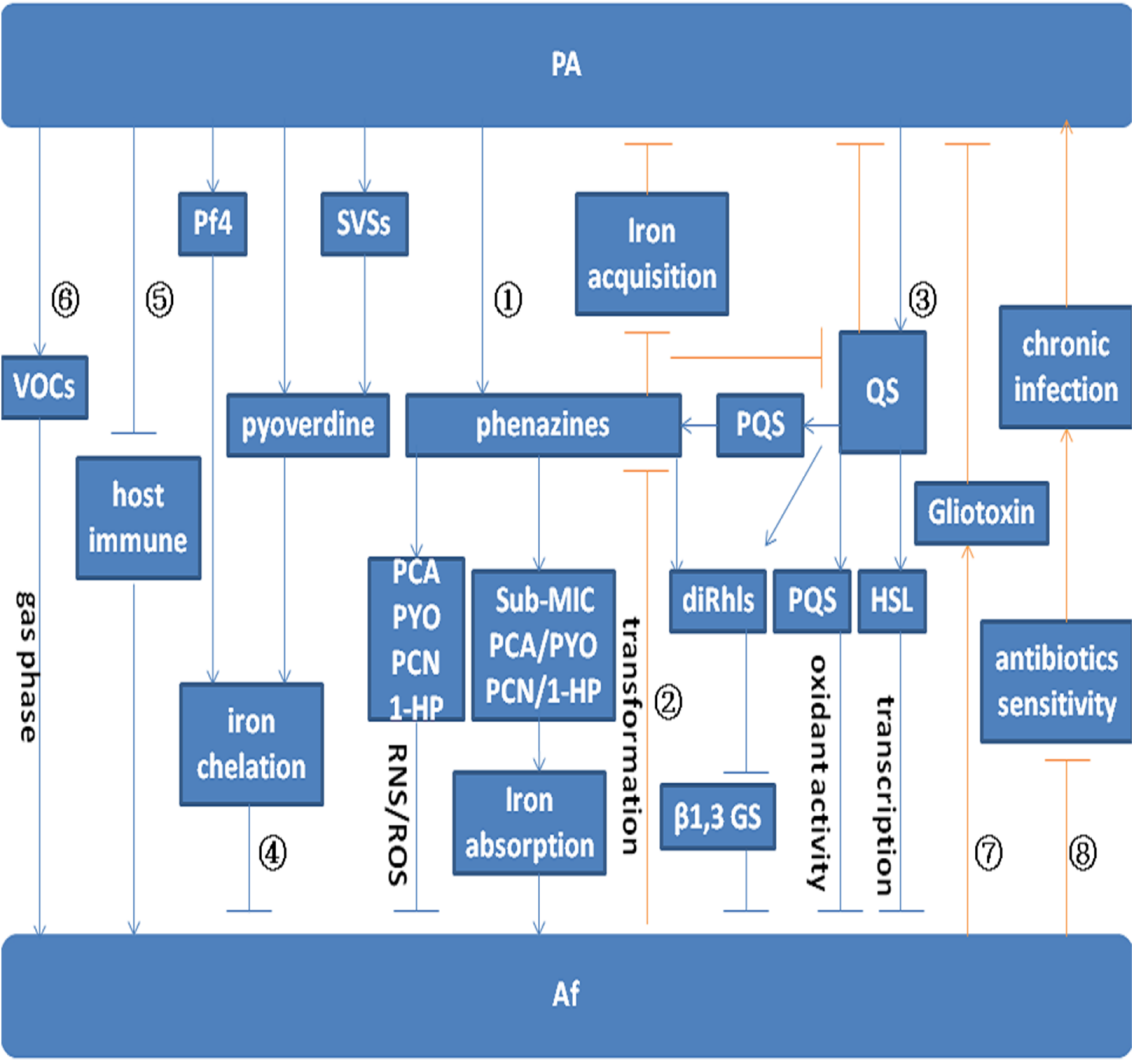
Model for the interaction between *P. aeruginosa* and *A. fumigatus.* Arrows indicate promotion. Arrows without heads indicate inhibition. Blue lines indicate the effect of *P. aeruginosa* on *A. fumigatus*. Red lines indicate the effect of *A. fumigatus* on *P. aeruginosa*. Pathway ① indicates the effect of *P. aeruginosa* on *A. fumigatus* by phenazine. PYO, PCA, PCN, and 1-HP inhibit *A. fumigatus* growth by inducing the production of ROS and RNS. Sub-MIC PYO, PCA, PCN, and 1-HP promote *A. fumigatus* growth by iron absorption. *A. fumigatus* growth can be inhibited by diRhls, which blocks β1,3 GS activity. Pathway ② shows the effect of *A. fumigatus* on *P. aeruginosa* by phenazine transformation. The metabolic conversion of phenazine by *A. fumigatus* inhibits the reduction of Fe^3+^ and affects QS system regulation in *P. aeruginosa*. Pathway ③ depicts the inhibition of toxic products and small molecules regulated by the QS system. The QS system in *P. aeruginosa* inhibits *A. fumigatus* growth via the effect of diRhls, PQS, and 3-oxo-C12 HSL. Pathway ④ shows that *P. aeruginosa* inhibits *A. fumigatus* growth via the effect of pyoverdine, Pf4, and SVSs on *A. fumigatus* iron deprivation. Pathway ⑤ and Pathway ⑥ illustrate *P. aeruginosa* promotion of *A. fumigatus* growth through the inhibition of host immune components and emission of VOCs. Pathway ⑦ shows that gliotoxin produced by *A. fumigatus* interferes with the metabolic growth of *P. aeruginosa*. Pathway ⑧ shows that *A. fumigatus* reduces the sensitivity of *P. aeruginosa* to antibiotics and promotes chronic infection.

*Pseudomonas aeruginosa*-secreted phenazines prevent the growth of *A. fumigatus*. It is thought that the toxic effects of phenazines on prokaryotes and diverse eukaryotic hosts result from their redox activities or inactivation of oxidative stress response proteins (Hassett et al., 1992; Muller, 2002; O’Malley et al., 2003). In target cells, reduced phenazines are oxidized by NAD(P)H and oxygen to generate reactive oxygen species (ROS), specifically O_2_^·−^ Moreover, generation of reactive nitrogen species (RNS) is induced by overproduction of O_2_^·−^ (Martínez & Andriantsitohaina, 2009). Nitric oxide (NO^·^) is produced by mitochondrial processes, and highly toxic peroxynitrite radicals (ONOO^−^) are generated via reactions between NO^·^ with O_2_^·−^ radicals (Martínez & Andriantsitohaina, 2009). Overall, mitochondria are the main target of phenazine-produced ROS and RNS, and phenazines have a significant impact on the mitochondrial ultrastructure of *A. fumigatus* hyphae. All four phenazines (PYO, PCA, PCN, 1-HP) show *A. fumigatus* growth inhibitory effects by inducing the production of ROS, specifically O_2_^·−^, and the RNS ONOO^−^ (pathway ① in Fig. 1) (Briard et al., 2015).

Another related study reported that phenazine-derived metabolites acting as interspecies signals can affect filamentous fungal development through oxidative stress regulation (Zheng et al., 2015b). In *P. aeruginosa*–*A. fumigatus* co-culture biofilms, development of the latter is differentially modulated by phenazine-derived metabolites of the former. With a decreasing phenazine gradient, *A. fumigatus* shifts from weak vegetative growth to an asexual sporulation phase (conidiation), and this shift in morphology is correlated with the production of phenazine radicals and concomitant ROS generation by phenazine redox cycling.

DiRhls induce *A. fumigatus* to produce an extracellular matrix that facilitates *P. aeruginosa* binding. *A. fumigatus* growth can be inhibited by diRhls, which blocks the β1,3 glucan synthase (GS) activity (pathway ① in Fig. 1) (Briard et al., 2017).

A recent study reported that a double phenazine mutant was similar to the wild-type organism in terms of its inhibitory power against *A. fumigatus* (Sass et al., 2018), with little difference caused by the complete lack of phenazine molecules due to mutation. The results of this study are different from those of previous studies. Several previous studies have emphasized the inhibitory power of phenazines on *A. fumigatus* (Briard et al., 2015, 2017; Zheng et al., 2015b), but a deficiency of these molecules via mutation appeared to cause little difference in this study. This finding suggests that the concentrations of phenazines that have been previously studied *in vitro* may be irrelevant to those in vivo. In addition, compensation for the loss of phenazine-mediated inhibitory activity by upregulation of other factors in the mutants could not be excluded in this study. Further research is needed to explore the effect of phenazines on the growth of *A. fumigatus*.

##### Effects of the inhibition of the QS system on the growth of A. fumigatus

The *P. aeruginosa* QS network plays a role in inhibiting *A. fumigatus* growth and biofilm formation (pathway ③ in Fig. 1) (Mowat et al., 2010). The las QS system is essential for the production of the diffusible signaling molecule acyl homoserine lactone (AHL) *N*-(3-oxododecanoyl)-l-homoserine lactone (3-oxo-C12 HSL) (Smith & Iglewski, 2003), resulting in the expression of specific target genes in *P. aeruginosa*. In addition, 3-oxo-C12 HSL is one of the AHLs frequently identified in extracts of respiratory secretions from CF patients infected with *P. aeruginosa* (Smith & Iglewski, 2003). By utilizing two *P. aeruginosa* QS knockout strains, PAO1:Δ*LasI* and PAO1:Δ*LasR*, one study illustrated that 3-oxo-C12 HSL inhibits *A. fumigatus* biofilm formation (Mowat et al., 2010). The PAO1:Δ*LasI* strain was unable to synthesize 3-oxo-C12 HSL, whereas PAO1:Δ*LasR* synthesized 3-oxo-C12 HSL but could not respond to it. Furthermore, *A. fumigatus* growth was significantly greater when in direct co-culture with PAO1:Δ*LasI* and PAO1:Δ*LasR* than in co-culture with wild-type PAO1. The indirect effect of the *P. aeruginosa* QS knockout strains on *A. fumigatus* biofilm development was assessed using the Transwell system, which showed significantly less inhibition of *A. fumigatus* biofilm development when in indirect co-culture with PAO1:Δ*LasI* and PAO1:Δ*LasR* than with the wild-type strain. Additionally, the cellular viability of *A. fumigatus* conidia and the biomass of *A. fumigatus* biofilms were reduced by diffusible and heat-stable soluble molecules, such as decanol, decanoic acid and dodecanol (structurally similar to the QS molecules produced by *P. aeruginosa*) in a concentration-dependent manner. At the molecular level, it is likely that these molecules lead to hyphal repression by affecting key transcription factors (Mowat et al., 2010).

In CF patients, *P. aeruginosa* produces rhamnolipids (Rhls), which are controlled by the QS system. Rhls are largely composed of diRhls and monorhamnolipids, and the diRhls secreted by *P. aeruginosa* may affect *A. fumigatus*. For example, diRhls induce *A. fumigatus* to produce an extracellular matrix that facilitates binding by *P. aeruginosa* (Briard et al., 2017). As stated above, diRhls also inhibit *A. fumigatus* growth by blocking β1,3 GS activity and altering cell wall architecture. In the presence of diRhls, *A. fumigatus* displays multibranched hyphae and a thicker cell wall rich in chitin. This growth phenotype of *A. fumigatus* is similar to that following treatment with anti-fungal echinocandins. Although the two rhamnose moieties attached to fatty acyl chains are essential structures for the interaction of diRhl with β1,3 GS, the site of β1,3 GS action differs between diRhls and echinocandins. Overall, diRhls and azole anti-fungals exhibit a synergistic anti-fungal effect (Briard et al., 2017).

A recent study reported that alkylhydroxyquinolones (AHQs), autoinducers secreted by *P. aeruginosa*, could suppress biofilm formation in *A. fumigatus*. The AHQ interkingdom signaling molecules 2-heptyl-3-hydroxy-4-quinolone (PQS) and 2-heptyl-4-quinolone (HHQ), which are involved in QS in *P. aeruginosa*, were both able to alter *A. fumigatus* biofilm biomass and structure (Reen et al., 2016). Both pro- and anti-oxidant activities have been reported for PQS and HHQ. AHQ interkingdom signaling molecules can interact with lipopolysaccharides, cellular membranes, and membrane vesicles in several bacterial species (Häussler & Becker, 2008). Redox-active phenazines of *P. aeruginosa*, which exhibit inhibitory activity against *A. fumigatus* growth, are also controlled by AHQs (Moree et al., 2012). Overall, these small interkingdom signaling molecules of *P. aeruginosa* disrupt *A. fumigatus* biofilm formation and render *A. fumigatus* susceptible to clearance by drugs. As these bacterial molecules are selectively non-cytotoxic to host cell lines, they may be used as viable molecular therapeutics.

The anti-*A. fumigatus* capacity of *P. aeruginosa* pyoverdine mutants has been assessed in recent studies. Some residual inhibition of *A. fumigatus* can be detected in pyoverdine mutants, and in addition to the anti-*Aspergillus* effect of pyoverdine, other inhibitors may contribute to the total fungal inhibition by wild-type *P. aeruginosa*. Some of these residual inhibitors in pyoverdine mutants may be related to QS-regulated metabolites, such as Rhls. The anti-*Aspergillus* ability of QS mutants was also examined, with the results showing that QS-regulated metabolites have an important anti-*Aspergillus* function. Indeed, these metabolites are potential intermicrobial inhibitors. The decreased anti-*A. fumigatus* activity of QS mutants might be related to loss of the combined activity of many downstream products, and decreases in pyoverdine production in QS mutants may also lead to their reduced anti-*Aspergillus* capacity (Sass et al., 2018).

##### Inhibition of A. fumigatus growth by Fe metabolism

*Pseudomonas aeruginosa* inhibits *A. fumigatus* growth through Fe limitation, which can, in part, result from the modulation of siderophore production by the fungus due to metabolites from the bacterium (Phelan et al., 2014).

Recent research has shown that *P. aeruginosa* pyoverdine can suppress *A. fumigatus* growth and biofilm formation via the chelation of iron, reducing its availability to *A. fumigatus* (pathway ④ in Fig. 1) (Sass et al., 2018). In this study, pvdD pchE and pvdD mutants (loss of pyoverdine and siderophore), which are defective in inhibiting *A. fumigatus* growth and biofilm formation in various assays, were evaluated. The inhibitory effect of pyoverdine deletion mutants was restored with pure pyoverdine, and the *A. fumigatus* sidA mutant that is unable to produce siderophores was found to be hypersusceptible to *P. aeruginosa* metabolites and to pyoverdine. Thus, the siderophore-deficient *A. fumigatus* mutant was readily inhibited by *P. aeruginosa*. Clinical *P. aeruginosa* isolates derived from the lungs of CF patients have revealed a correlation between the amount of pyoverdine produced and the anti-fungal activity of clinical samples. The results suggest that the siderophore pyoverdine is an important inhibitory molecule (Sass et al., 2018).

As pyoverdine can capture iron from the environment, it can deprive *A. fumigatus* of the iron that is essential for its growth and metabolism. Iron sequestration by pyoverdine leads to iron starvation and increased siderophore secretion by *A. fumigatus* (Sass et al., 2018). In a shared microenvironment, *P. aeruginosa* and *A. fumigatus* compete for iron to promote their own survival, and high pyoverdine expression antagonizes *A. fumigatus* metabolism and growth, which might support anti-fungal treatment. Key aspects of the competition between *P. aeruginosa* and *A. fumigatus* include the relative amounts of siderophores produced, the speed of siderophore production, and the relative affinity for Fe.

##### Bacteriophage Pf4 inhibits the metabolic activity of A. fumigatus biofilms

**Figure 2:**
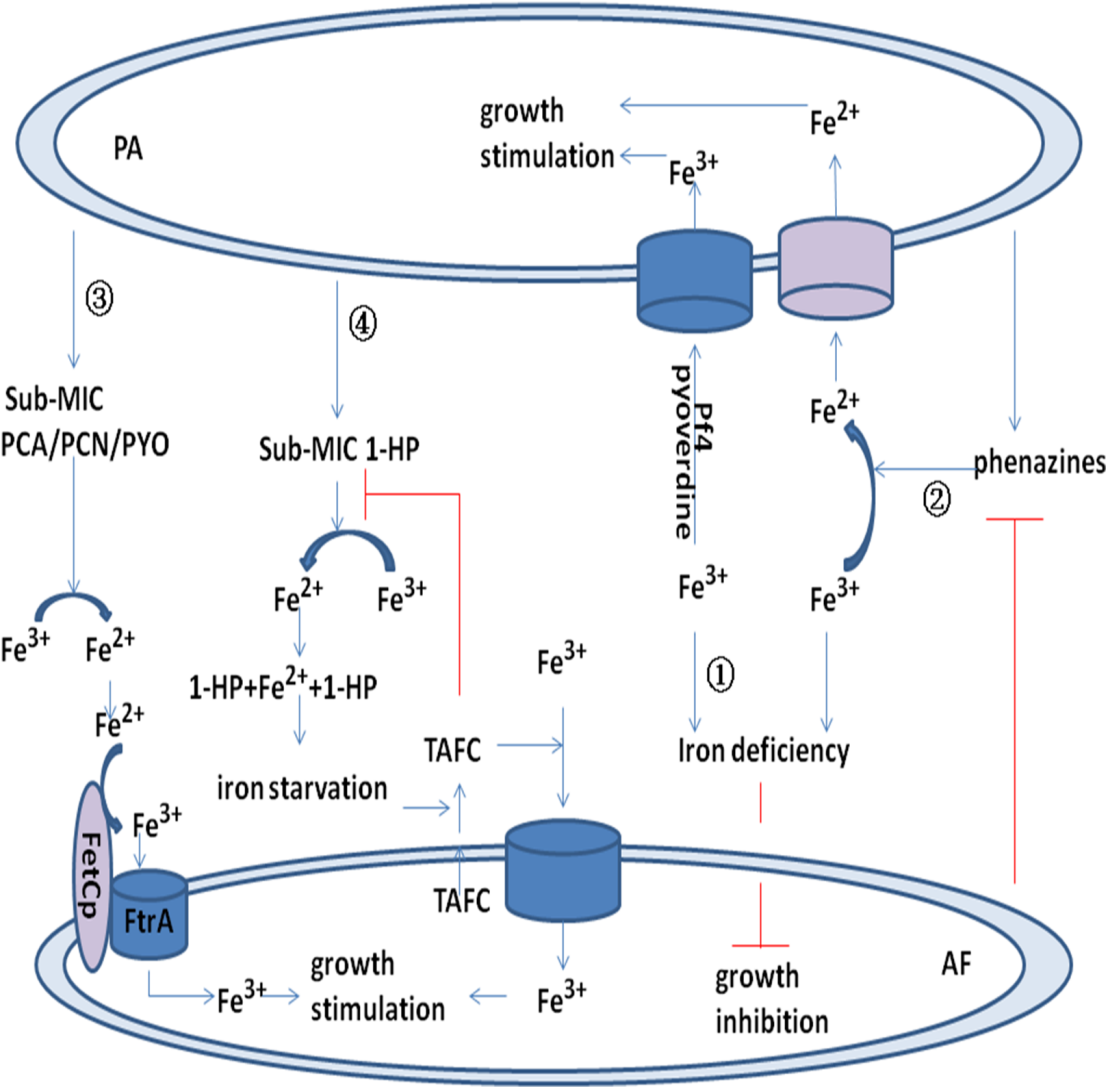
Model for the interaction between *P. aeruginosa* and *A. fumigatus* by iron uptake and competition. Pyoverdine and Pf4 phage bind to Fe^3+^ and promote uptake by *P. aeruginosa*. Pyoverdine and Pf4 phage deprive *A. fumigatus* of Fe^3+^ and inhibit its growth (pathway ①). Phenazine reduces Fe^3+^ to Fe^2+^ and promotes *P. aeruginosa* uptake of Fe^2+^. Phenazine is converted by *A. fumigatus* into metabolic products with potentially modified redox potentials. These products may inhibit the reduction of Fe^3+^ in *P. aeruginosa* (pathway ②). Sub-MIC PYO, PCN, and PCA reduce Fe^3+^ to Fe^2+^ and promote the FetCp/FtrA complex of *A. fumigatus* to take up Fe^2+^ (pathway ③). Sub-MIC 1-HP reduces Fe^3+^ to Fe^2+^, and two 1-HP molecules bind the newly formed Fe^2+^. This chelating activity induces iron starvation and activates triacetylfusarinine C (TAFC). TAFC promotes *A. fumigatus* uptake of Fe^3+^ and stimulates its growth (pathway ④).

In a recent study, it was reported that the Pf4 phagosome can inhibit *A. fumigatus* metabolism and growth by binding iron and causing iron deficiency (pathway ④ Fig. 1) (Penner et al., 2016). Pf4 inhibition of *A. fumigatus* is caused by iron binding and the sequestration of *A. fumigatus* iron resources (pathway ① Fig. 2), and inhibition of *A. fumigatus* metabolism by Pf4 can be overcomed with ferric iron supplementation. Moreover, inhibition of *A. fumigatus* biofilm formation by phages is reversed by low doses of iron, indicating that *A. fumigatus* is more sensitive to iron inhibition by the Pf4 phage than it is to other elements in *P. aeruginosa* supernatants (Penner et al., 2016). This Pf4 phage-mediated inhibition was found to be dose dependent and could be alleviated by phage denaturation. This inhibition of Pf4 was more significant in preformed *A. fumigatus* biofilms than during biofilm formation. In contrast, Pf4 had no effect on planktonic conidia (Penner et al., 2016). These findings suggest that the site of phage action is specific to the extracellular matrix or hyphae (Reichhardt et al., 2015). Another two phages, Pf1 and fd, showed no inhibitory action against *A. fumigatus*. Pf4 attaches to *A. fumigatus* hyphae, and fungal inhibition may occur at the biofilm surface. The shorter phage Pf1 did not bind as extensively to *A. fumigatus* biofilms as did Pf4 and exhibited less efficient inhibition (Penner et al., 2016).

Acute infection of *P. aeruginosa* by Pf bacteriophage can decrease the production of pyoverdine and the inhibitory capacity toward *A. fumigatus* biofilms. Thus, the reduced production of anti-microbials by *P. aeruginosa* infected by Pf bacteriophage may promote co-infection with *A. fumigatus* in CF airways (Secor et al., 2017).

##### SCVs in A. fumigatus intermicrobial competition

In a study of SCVs in intermicrobial competition with *A. fumigatus* (Anand et al., 2017b), the SCVs isolated from *P. aeruginosa* was shown to inhibit *A. fumigatus* biofilm formation, and this inhibitory capacity toward *A. fumigatus* biofilm was found to be related to pyoverdine (pathway ④ in Fig. 1) (Anand et al., 2017b). Indeed, isolated SCVs with high pyoverdine production had the highest inhibitory capacity in every co-culture method evaluated. Correspondingly, the two SCV isolates with the lowest inhibitory activities did not produce pyoverdine, suggesting that pyoverdine is the key *P. aeruginosa* inhibitor of *A. fumigatus* (Anand et al., 2017b).

*Pseudomonas aeruginosa* SCVs exhibit heterogeneity in inhibiting *A. fumigatus* biofilms. For instance, the inhibitory abilities of clinical SCVs isolates and reference CF non-mucoid isolates of *P. aeruginosa* or filtrates from *P. aeruginosa* planktonic or biofilm cultures were compared by coincubation with *A. fumigatus* during biofilm formation or in preformed biofilm. The metabolic activities of *A. fumigatus* biofilms were measured by different assays, with pyoverdine in filtrates being measured by spectrophotometry. The results showed that SCVs inhibited *A. fumigatus* biofilm formation, although the inhibitory effects of different SCVs were quite different. By adjusting planktonic culture filtrates, differences in SCV inhibition were related to SCV growth or deficient inhibitor production. Overall, the ability of SCVs to inhibit *A. fumigatus* biofilm was related to pyoverdine (Anand et al., 2017b). Thus, SCVs isolated from *P. aeruginosa* may be important in CF because they are capable of inhibiting *A. fumigatus* biofilm.

##### Inhibition of A. fumigatus by P. aeruginosa under conditions of hypoxia

Most studies examining the inhibition of *A. fumigatus* by *P. aeruginosa* have been performed under normoxic conditions. However, patients with acute exacerbation or progression of CF may exhibit hypoxia in focal lung sites (Cowley et al., 2015; Lambiase, Catania & Rossano, 2010; Worlitzsch et al., 2002). *A. fumigatus* inhibition by *P. aeruginosa* was recently evaluated under hypoxic conditions (Anand, Clemons & Stevens, 2017a), and the results showed that although the inhibitory activities of *P. aeruginosa* were effective under aerobic, hypoxic, or anaerobic conditions, *P. aeruginosa* growth was slow under hypoxic or anaerobic conditions, thus decreasing the ability of *P. aeruginosa* filtrates to inhibit *A. fumigatus* growth and biofilm formation. Regardless of the planktonic or biofilm state, the extracellular molecules produced by *P. aeruginosa* under anaerobic conditions were less inhibitory toward *A. fumigatus* growth and biofilm formation than were those under aerobic conditions. Therefore, the inhibitory power of *P. aeruginosa* against both *A. fumigatus* preformed biofilm and biofilm formation was decreased under hypoxic conditions (Anand, Clemons & Stevens, 2017a).

During the course of CF progression, *P. aeruginosa* mutants with a low level of pyoverdine production often appear, and the ratio of Fe^3+^ to Fe^2+^ decreases under hypoxic conditions. Intracellular iron acquisition by *P. aeruginosa* occurs mainly through the ingestion of low-activity Fe^2+^ via the production of phenazines and membrane permease (Cartron et al., 2006; Cornelis & Dingemans, 2013; Nguyen & Oglesby-Sherrouse, 2015). *P. aeruginosa* in the airway of CF patients typically shows low pyoverdine expression, and as mentioned above, pyoverdine can inhibit *A. fumigatus* growth by depriving *A. fumigatus* of iron. Under hypoxic conditions, the inhibitory effect of *P. aeruginosa* on *A. fumigatus* declines; thus, the growth of *A. fumigatus* is promoted (Sass et al., 2018). These findings explain why *A. fumigatus* is able to colonize CF airways following *P. aeruginosa* colonization and why *A. fumigatus* may persist during CF disease progression or chronic lung infection (Amin et al., 2010; Baxter et al., 2013a; Fillaux et al., 2012; Forsyth et al., 1988; Mirković et al., 2016; Nicolai et al., 1990; Speirs, Van Der Ent & Beekman, 2012).

After a long period of hypoxia, the interaction between *P. aeruginosa* and *A. fumigatus* appears to be similar to that under normoxic conditions (Anand, Clemons & Stevens, 2017a). Other factors may also affect *P. aeruginosa* and *A. fumigatus* interactions, such as prolonged use of antibiotics or inhaled corticosteroids (Noni et al., 2014).

##### The different inhibitory capacities of P. aeruginosa on A. fumigatus between planktonic and biofilm states

*Pseudomonas aeruginosa* and *A. fumigatus* are commonly found in the airways of patients with CF in the form of biofilms, and their pathogenicity and resistance in the biofilm state differ from those in the planktonic state. The inhibitory capacity of *P. aeruginosa* toward *A. fumigatus* in the biofilm state is also different from that in the planktonic state (Mowat et al., 2010). As an example, the supernatant extracted from *P. aeruginosa* biofilm was more effective than that extracted from planktonic cells (Ferreira et al., 2015). Pyoverdine plays an important role in *P. aeruginosa* biofilm formation, and its production is higher in biofilm than in planktonic cells (Visaggio et al., 2015). Thus, the *P. aeruginosa* biofilm-mediated suppression of *A. fumigatus* growth and biofilm formation is greater than planktonic *P. aeruginosa*-mediated suppression. It is also possible that other inhibitors may play an important role in this process (Anand et al., 2017b).

As mentioned above, the inhibitory effects of *P. aeruginosa* on *A. fumigatus* biofilm formation and preformed biofilms are different; it has been reported that *P. aeruginosa* can inhibit *A. fumigatus* biofilm formation but has almost no effect on preformed biofilms. The mature filamentous biofilms of *A. fumigatus* clearly restricted the inhibitory capacity of *P. aeruginosa* (Mowat et al., 2010), and another study showed that preformed *A. fumigatus* biofilm was more resistant to *P. aeruginosa* (Ferreira et al., 2015). According to a recent study, preformed *A. fumigatus* biofilms are inhibited by biofilm filtrates of *P. aeruginosa* strains isolated from CF patients via apoptosis, an effect that is related to mitochondrial membrane damage caused by metacaspase activation (Shirazi et al., 2016). In contrast, the inhibitory capacity of *P. aeruginosa* Pf4 phage toward *A. fumigatus* preformed biofilm is higher than that during biofilm formation. The *P. aeruginosa* phage Pf4 had little effect on planktonic conidial growth (Penner et al., 2016), suggesting that hyphae or the extracellular matrix is the specific site of phage action (Reichhardt et al., 2015).

##### The different inhibitory abilities of P. aeruginosa on A. fumigatus conidia and hyphae

After *A. fumigatus* conidia colonization in the CF patient airway, *A. fumigatus* gradually forms a biofilm that is rich in hyphae, and *P. aeruginosa* exhibits different inhibitory capacities toward *A. fumigatus* conidia and hyphae. In simultaneous static co-cultures, *P. aeruginosa* cells can effectively kill *A. fumigatus* conidia cells, but *P. aeruginosa* cells show only a minor inhibitory effect on sporelings grown for 12 h or longer as well as hyphae (Manavathu, Vager & Vazquez, 2014). Indeed, during co-cultivation with *P. aeruginosa*, *A. fumigatus* sporelings grown for 12 h or longer and young hyphae were stronger than ungerminated conidia with respect to the formation of *P. aeruginosa*–*A. fumigatus* biofilm.

*Aspergillus fumigatus* hyphae can withstand the fungicidal effect of *P. aeruginosa* and can produce the cytotoxic compound gliotoxin, which has anti-bacterial activity. Production of mycotoxin increases during mycelial growth and biofilm formation in *A. fumigatus* (Manavathu, Vager & Vazquez, 2014), and *P. aeruginosa* growth and its ability to kill *A. fumigatus* are suppressed with increasing levels of gliotoxin. In addition, virulence factor production and the inhibitory action of *P. aeruginosa* are strengthened by this increased metabolic activity in cells. Overall, the metabolic activity of germinating conidia and young sporelings is strong, whereas that of mature hyphae is limited in the apical regions of filaments. The apex, which has high metabolic activity, is the site at which *P. aeruginosa* binds to *A. fumigatus* hyphae and acquires nutrients (Toljander et al., 2007); hyphae, which has low metabolic activity, are not sensitive to the toxic molecules of *P. aeruginosa*. In fact, the cell walls of mature hyphae are poorly permeable to the toxic molecules of *P. aeruginosa*. Hence, mature hyphae are not easily killed by *P. aeruginosa* (Manavathu, Vager & Vazquez, 2014).

##### Comparison of the inhibitory effect between different P. aeruginosa strains from CF patients and non-CF patients

In addition to CF patients, *P. aeruginosa* and *A. fumigatus* also co-exist in the airways of patients with conditions such as chronic obstructive pulmonary disease, bronchiectasis, and hospital-acquired pneumonia. *P. aeruginosa* in CF patient airways can be divided into two types: mucoid and non-mucoid. A recent survey showed that both non-CF and CF *A. fumigatus* strains are inhibited by *P. aeruginosa* metabolic products (Nazik et al., 2017), and another study reported that both *P. aeruginosa* cells and filtrates isolated from CF patients had greater inhibitory effects on *A. fumigatus* growth and biofilm formation *in vitro* than did materials isolated from non-CF patients (Ferreira et al., 2015). Furthermore, non-mucoid *P. aeruginosa* exerted greater inhibitory effects on *A. fumigatus* than did mucoid *P. aeruginosa* in CF patients (Shirazi et al., 2016).

*Pseudomonas aeruginosa* isolated from the airway of CF patients has a greater chance of contact and longer duration of co-existence with *A. fumigatus* than isolates from non-CF patients. In addition, *P. aeruginosa* isolated from CF patients produces more toxic products and inhibitors than that from non-CF patients. Thus, CF patient-derived *P. aeruginosa* has a greater inhibitory effect against *A. fumigatus* growth and biofilm formation than strains isolated from non-CF patients. Mucoid *P. aeruginosa* usually exists in the deep and hypoxic zone of the lung (Gaspar et al., 2013; Pressler et al., 2006; Tramper-Stranders et al., 2012), and the synthesis of toxic products and inhibitors of *P. aeruginosa* is reduced under hypoxic conditions. Overall, the inhibitory capacity of mucoid CF *P. aeruginosa* filtrates is less than that of non-mucoid CF filtrates.

#### Promotion of A. fumigatus growth by P. aeruginosa

*Pseudomonas aeruginosa* induces *A. fumigatus* growth through the action of sub-bacteriostatic concentrations of phenazines and volatile organic compounds (VOCs). Pathogen reproduction requires iron ions, and phenazines promote Fe^3+^ reduction in CF patients infected with *P. aeruginosa* (Hunter et al., 2013). In the early stages of infection, host immune cell molecules, such as lactoferrin or transferrin, actively chelate Fe^3+^ ions and inhibit the growth of *P. aeruginosa* and *A. fumigatus*. However, phenazines can promote *P. aeruginosa* and *A. fumigatus* growth by iron acquisition, and *P. aeruginosa* phenazines can reduce Fe^3+^ to Fe^2+^ and liberate Fe^3+^ from host immune cells (Banin, Vasil & Greenberg, 2005; Hernandez, Kappler & Newman, 2004; Wang et al., 2011). *P. aeruginosa* produces VOCs during the course of infection and reproduction, and these molecules can be detected in sputum samples of CF patients infected with *P. aeruginosa* (Goeminne et al., 2012).

##### Promotion effect of P. aeruginosa on A. fumigatus colonization and growth

Under certain conditions, *P. aeruginosa* promotes the growth of *A. fumigatus*. Therefore, many CF patients are susceptible to infection with *A. fumigatus* after infection with *P. aeruginosa* (Paugam et al., 2010). One study hypothesized that *P. aeruginosa* infection promotes the evolution of *A. fumigatus* sensitization (Kraemer et al., 2006).

Another study reported reduced colonization of *A. fumigatus* after anti-infective treatment of *P. aeruginosa* in patients with acute exacerbation of CF, potentially because the bacterium protects *A. fumigatus* via immune factors and growth conditions (Baxter et al., 2013b). During acute exacerbation in CF patients, the colonization and growth of *A. fumigatus* may be related to the negative effects of *P. aeruginosa* on host lung function and immune defense. Resistance of *P. aeruginosa* biofilms to host immune responses contributes to the growth and multiplication of *A. fumigatus* (pathway ⑤ in Fig. 1) (Baxter et al., 2013a). By analyzing pre- and post-antibiotic sputum samples from adult CF patients, a study showed that intravenous antibiotics targeting *P. aeruginosa* during CF pulmonary exacerbations had a negative impact on the colonization and growth of *A. fumigatus* (Baxter et al., 2013b). Because *P. aeruginosa* contributes to the colonization and growth of *A. fumigatus* in CF patients, it is also possible that both microbes work together to combat host immune factors, resulting in increased infection and decreased pulmonary function. Nonetheless, their relationship may become competitive with growth, and *P. aeruginosa* inhibits *A. fumigatus* in various ways, as described above. Further research is required to verify the interdependence between these two organisms for survival within the airways of CF patients.

##### Sub-bacteriostatic concentrations of phenazines induce A. fumigatus growth

Regarding *P. aeruginosa* and *A. fumigatus* competitive growth, the former produces phenazines to inhibit the growth of the latter (Zheng, Keller & Wang, 2015a), yet one study demonstrated that sub-bacteriostatic concentrations of phenazines can induce *A. fumigatus* growth in specific situations. For instance, it was reported that sub-bacteriostatic concentrations of PYO, PCA, and PCN can induce *A. fumigatus* growth by promoting iron uptake. The redox function of 1-HP, which is capable of chelating iron ions and inducing iron starvation in *A. fumigatus*, can also promote *A. fumigatus* growth (pathway ① in Fig. 1) (Briard et al., 2015).

*Aspergillus fumigatus* can obtain iron resources through low-affinity ferrous iron uptake, high-affinity reductive iron uptake and siderophore-mediated iron uptake (Schrettl et al., 2004). Three sub-bacteriostatic phenazines, PYO, PCA, and PCN, can induce the growth of an *A. fumigatus* mutant lacking *SidAp* and siderophore biosynthesis under iron starvation conditions, and it was suggested that phenazines reduce Fe^3+^ to Fe^2+^ and promote the ferroxidase FetCp/permease FtrAp complex of the *A. fumigatus* mutant to take up Fe^2+^ (pathway ③ in Fig. 2) (Briard et al., 2015). Fusarinine C (FsC) and triacetylfusarinine C (TAFC) are two extracellular siderophores of *A. fumigatus*, while ferricrocin and hydroxyferricrocin are two intracellular siderophores of *A. fumigatus* (Haas, 2012). Sub-bacteriostatic 1-HP produced by *P. aeruginosa* promotes *A. fumigatus* growth by iron chelation and stimulation of TAFC secretion (pathway ④ in Fig. 2) (Briard et al., 2015).

##### VOCs promote A. fumigatus growth

A recent study showed that VOCs released by *P. aeruginosa* can promote *A. fumigatus* growth (pathway ⑥ in Fig. 1) (Briard, Heddergott & Latgé, 2016), and dimethyl sulfide was found to be a VOC with an enhancement effect on *A. fumigatus*, which is mediated by the gas phase. During sulfur starvation, *A. fumigatus* utilizes exogenous VOCs to promote growth, and in patients with CF, it is possible that *P. aeruginosa* promotes the colonization and growth of *A. fumigatus* by releasing VOCs, causing a rapid decline in lung function (Briard, Heddergott & Latgé, 2016). This result is consistent with the results of past clinical studies showing that *P. aeruginosa* and *A. fumigatus* co-infection resulted in decreased lung function (Amin et al., 2010; Baxter et al., 2013b).

Without direct contact, *P. aeruginosa* and *A. fumigatus* interact through signaling molecules such as VOCs. With direct contact, *P. aeruginosa* can release the corresponding signaling molecules to promote *A. fumigatus* colonization and growth. However, when these pathogenic microorganisms grow and contact one another, they exert a mutual inhibitory effect with regard to nutrient competition (Briard, Heddergott & Latgé, 2016).

#### The effect of A. fumigatus on P. aeruginosa

*Pseudomonas aeruginosa* and *A. fumigatus* interact with each other under co-growth conditions in patients with CF and other chronic pulmonary infection diseases. *A. fumigatus* can also resist inhibition by *P. aeruginosa* and, to a certain extent, affect its growth and metabolism.

##### Metabolic transformation of phenazines produced by P. aeruginosa

Phenazines inhibit *A. fumigatus* by inducing the production of ROS and RNS. Sod2p of *A. fumigatus* can resist the injury caused by ROS and RNS and antagonize inhibition by *P. aeruginosa* (Briard et al., 2015). Another study demonstrated that the phenazines produced by *P. aeruginosa* can be metabolically converted by *A. fumigatus* to reduce their inhibitory effect. As an example, PCA can be converted to 1-HP,1-methoxyphenazine and phenazine-1-sulfate. Although 1-HP has an inhibitory effect on *A. fumigatus*, 1-HP was also able to induce the production of the *A. fumigatus* siderophores TAFC and FsC (Moree et al., 2012). Regardless, previous experiments have shown that 1-HP inhibits bacterial siderophore biosynthesis (Dietrich et al., 2008). PCA induces the reduction of Fe^3+^ to Fe^2+^ for *P. aeruginosa* biofilm formation (Wang et al., 2011), and conversion of PCA by *A. fumigatus* may also decrease *P. aeruginosa* iron acquisition for metabolism and biofilm formation. Phenazine is converted by *A. fumigatus* into metabolic products with potentially altered redox potentials, and these products may inhibit Fe^3+^ reduction in *P. aeruginosa* (pathway ② Fig. 2). The phenazines PYO and PCA produced by *P. aeruginosa* can be converted to phenazine dimers by *A. fumigatus*, which have a decreased inhibitory effect on *A. fumigatus*. The QS signaling molecule PYO of *P. aeruginosa* affects transcriptional regulation and induces biofilm formation. Metabolic conversion of PYO by *A. fumigatus* might have an effect on QS system regulation. Thus, *A. fumigatus* can transform *P. aeruginosa* metabolites and radically alter the effects on their interaction, including the degree of inhibition (pathway ② in Fig. 1) (Moree et al., 2012).

##### Gliotoxin produced by A. fumigatus interferes with the metabolic growth of P. aeruginosa

During co-culture, *A. fumigatus* can also invoke its own metabolism and signaling molecules to disrupt *P. aeruginosa* growth. Gliotoxin produced by *A. fumigatus* is also a major immunoevasive toxin that is important in mediating *A. fumigatus*-associated colonization within the context of CF (Chotirmall et al., 2014). Overall, the gliotoxin secreted by *A. fumigatus* suppresses the inhibitory ability and growth of *P. aeruginosa* (pathway ⑦ in Fig. 1) (Manavathu, Vager & Vazquez, 2014).

##### Reducing the sensitivity of P. aeruginosa to antibiotics and promoting chronic infection

Under specific conditions, *A. fumigatus* can maintain the growth of *P. aeruginosa* in a co-existence scenario. One case-control study showed that CF patients infected by *A. fumigatus* could easily develop chronic *P. aeruginosa* infections despite receiving anti-microbial therapy (pathway ⑧ in Fig. 1) (Pressler et al., 2006).

The sensitivity of *P. aeruginosa* to antibiotics also changes when it co-exists with *A. fumigatus*. One *in vitro* study showed that *P. aeruginosa* in a polymicrobial biofilm with *A. fumigatus* was less susceptible to cefepime than *P. aeruginosa* in a monomicrobial biofilm state (pathway ⑧ in Fig. 1) (Manavathu, Vager & Vazquez, 2014). The extracellular matrix of polymicrobial biofilms differs for *P. aeruginosa* monomicrobial biofilms, and the sensitivity of *P. aeruginosa* in polymicrobial biofilm to some antibiotics decreases as a result of the change in the biofilm extracellular matrix. Indeed, some antibiotics cannot kill *P. aeruginosa* in biofilm due to altered permeability of the polymicrobial biofilm extracellular matrix. In contrast, the change in extracellular matrix between polymicrobial biofilms and *A. fumigatus* monomicrobial biofilms is not obvious and may not be sufficient to cause changes in the sensitivity of *A. fumigatus* to anti-fungal agents. For example, the sensitivity of *A. fumigatus* to anti-fungal drugs, such as voriconazole and posaconazole, did not change in polymicrobial or monomicrobial biofilms (Manavathu, Vager & Vazquez, 2014).

At present, only a few studies have examined the effect of *A. fumigatus* on *P. aeruginosa*, and the underlying mechanism should be the focus of further research.

## Conclusion

In the co-infection state, *P. aeruginosa* interacts with *A. fumigatus* in a number of ways. In the early stage of CF, *P. aeruginosa* first colonizes and then grows, providing favorable nutritional and immunological conditions for infection and colonization by *A. fumigatus. P. aeruginosa* and *A. fumigatus* in the CF patient airway then promote each other’s growth and grow together. As the disease progresses and resources become less abundant in the co-existence environment, the interaction between *P. aeruginosa* and *A. fumigatus* shifts to mutual inhibition. It is suggested that antibiotic treatment against *P. aeruginosa* can inhibit the progression of *A. fumigatus* infection early during co-infection. When the infection is exacerbated and the condition of the CF patients deteriorates, anti-infective treatment against one pathogen may lead to the growth and reproduction of the other pathogen. At present, combined anti-infective therapy against both pathogens should be used, as the above possibilities need to be further investigated by a large number of clinical studies. The interaction between the two pathogens is quite complicated in the process of CF disease development, and many mechanisms of action remain unclear. Further studies examining the influence of *A. fumigatus* on *P. aeruginosa* and the immunomodulatory mechanism between these pathogens and the human body must be carried out to facilitate the treatment of CF patients with polymicrobial infections.

## Additional Information and Declarations

### Data Availability

The following information was supplied regarding data availability: The research in this article did not generate any data or code. This is a literature review.
